# Control of Several Emissions during Olive Pomace Thermal Degradation

**DOI:** 10.3390/ijms151018349

**Published:** 2014-10-13

**Authors:** Teresa Miranda, Sergio Nogales, Silvia Román, Irene Montero, José Ignacio Arranz, Francisco José Sepúlveda

**Affiliations:** 1 Department of Mechanical, Energetic and Materials Engineering, Industrial Engineering School, University of Extremadura, Avda Elvas s/n, 06071 Badajoz, Spain; E-Mails: senogales@alumnos.unex.es (S.N.); imontero@unex.es (I.M.); jiarranz@unex.es (J.I.A.); fsepulveda@unex.es (F.J.S.); 2 Department of Applied Physics, Industrial Engineering School, University of Extremadura, Avda Elvas s/n, 06071 Badajoz, Spain; E-Mail: sroman@unex.es

**Keywords:** combustion, emissions, biomass, mass spectrometry, thermogravimetry

## Abstract

Biomass plays an important role as an energy source, being an interesting alternative to fossil fuels due to its environment-friendly and sustainable characteristics. However, due to the exposure of customers to emissions during biomass heating, evolved pollutants should be taken into account and controlled. Changing raw materials or mixing them with another less pollutant biomass could be a suitable step to reduce pollution. This work studied the thermal behaviour of olive pomace, pyrenean oak and their blends under combustion using thermogravimetric analysis. It was possible to monitor the emissions released during the process by coupling mass spectrometry analysis. The experiments were carried out under non-isothermal conditions at the temperature range 25–750 °C and a heating rate of 20 °C·min^−1^. The following species were analysed: aromatic compounds (benzene and toluene), sulphur emissions (sulphur dioxide), 1,4-dioxin, hydrochloric acid, carbon dioxide and nitrogen oxides. The results indicated that pollutants were mainly evolved in two different stages, which are related to the thermal degradation steps. Thus, depending on the pollutant and raw material composition, different emission profiles were observed. Furthermore, intensity of the emission profiles was related, in some cases, to the composition of the precursor.

## 1. Introduction

Biomass is an important alternative to fossil fuels, and in fact, its use might be applied to many materials, such as woody biomass or algae [1], and its real implementation is being already considered [2,3,4]. Currently, the disposal of waste products is a problem in industrial and agricultural activities. In certain cases, they cause environmental pollution, so require recycling and, therefore, additional cost. Using biomass as an energy source helps to solve this problem, providing an added value to these materials (that is, energy) in a clean way. This possibility has raised the interest of researchers, using many materials that are considered waste products in order to obtain energy [5], applying different techniques [6].

The combustion process involves emissions that are considerably lower than those released during fossil fuel combustion, but some concerns should be taken into account: (a) many households and companies use biomass as the main energy source, especially in developing countries; (b) their exposure to pollutants is relatively constant; (c) steps in order to reduce this exposure are not always enough. This makes it necessary to perform studies that investigate the chemical processes taking place during biomass thermal decomposition, and check the emissions released during the process [7,8]. The control of emissions released during thermo–chemical degradation of biomass is a growing issue which has attracted the scientific community [5,6,7,8,9,10,11,12,13]. In these cases, some techniques, such as thermogravimetry (TG) and mass spectrometry (MS), are really important in order to assess the intensity of the emissions and their relation with mass loss. Indeed, TG–MS systems are quite useful and show immediate emissions from mass loss in biomass [14].

Some of the most worrying emissions related to biomass are aromatics compounds (such as benzene, toluene or phenol), sulphur, nitrogen (such as nitrogen oxides), chlorine compounds, dioxins and furans, which are considered dangerous to either the environment and/or human health [12]. However, the fact that those emissions evolve during heating must be taken into account.

There is evidence that sulphur content in raw material causes the emission of sulphur compounds; in the same way nitrogen and chlorine content produces significant releases in nitrogen oxides and dioxins, respectively [7], along with some indirect compounds contributing to acid rain. In previous studies, remarkable biomass heterogeneity was demonstrated when it comes to composition [13,15]. Namely, agricultural wastes, such as olive pomace (OP), were proved to show higher emissions when used as biomass fuels, on account of their proximate and ultimate composition (with higher N, S or Cl content compared to woody biomass). Moreover, there were clear differences between agricultural wastes and woody biomass when ash composition was studied [13].

As some emissions associated to olive pomace thermal degradation are related to its composition, one interesting way to lower this proportion could be to make mixtures of olive pomace with other biomass residues with lower content of some elements such as nitrogen.

The use of mixtures of different feedstock with the aim of improving certain characteristics (such as reducing emissions) during its exploitation has successfully been applied by Chagger *et al*. [11], Skodras *et al*. [16], Ulloa *et al*. [17], Idris *et al*. [18], and Menya *et al*. [19].

With the aim of decreasing some precursors (such as sulphur, nitrogen and chlorine), pyrenean oak (PK) was chosen to make blends with OP. Thus, by combining OP with PK, the emissions containing the above mentioned elements would be expected to be lower in the blends.

The aim of this work was to study the combustion of a series of pellets with variable proportions (0%, 25%, 50%, 75% and 100%), of pyrenean oak and olive pomace regarding their thermogravimetric analyses and determining the emissions associated to the following compounds: aromatic compounds: (a) benzene (C_6_H_6_) and toluene (C_7_H_8_); (b) sulphur compounds: sulphur dioxide (SO_2_); (c) dioxins: 1,4-dioxin (C_4_H_4_O_2_); (d) hydrochloric acid (HCl), carbon dioxide (CO_2_) and nitrogen oxides (NO_x_). These results were compared to biomass composition (proximate, ultimate analysis and ash composition).

## 2. Results and Discussion

Table 1 shows the proximate, ultimate and high heating value (HHV) analyses of the various blends in study. As it can be observed, all blends presented low S quantities, which is advantageous since it minimizes corrosion problems related to acid formation and therefore prevents acid rain. However, it is noticeable that increasing OP content is related to a higher nitrogen proportion. For sulphur, carbon and hydrogen content, no clear differences were observed.

**Table 1:** Proximate, ultimate and high heating value (HHV) analysis of blends.

Olive Pomace (%)	100	75	50	25	0
**Ultimate (%)**					
C *	45.25	45.56	45.76	45.50	45.69
N	1.92	1.33	0.99	0.71	0.58
H	6.14	6.10	6.13	6.02	6.12
S	0.15	0.09	0.08	0.09	0.13
**Proximate**					
Moisture **	6.86	7.65	7.40	5.54	4.70
Ash	5.55	5.40	5.02	4.62	3.71
Fixed carbon	17.3	17.1	17.6	16.3	12.7
Volatiles	77.2	77.5	77.4	79.1	83.6
**HHV (kcal/kg)**	5263	5043	4747	4596	4569

* dry basis; ** wet basis.

Table 2 shows Cl, Na and K content. As it can be observed, all the elements in the study increased as Olive Pomace content increased. Additionally, for pyrenean oak (OP0), Na content was not detected. In this case, Cl, Na and K content increased proportionally with a higher Olive Pomace proportion.

**Table 2:** Cl, Na and K content (%, wet basis).

Sample	Cl	Na	K
OP100	0.299	0.103	1.532
OP75	0.238	0.086	1.430
OP50	0.167	0.056	1.007
OP25	0.081	0.023	0.620
OP0	0.014	N.D.	0.264

N.D., not detected.

Ash composition was determined in order to study the effect of mineral matter on the samples and their thermal behavior. Table 3 shows the percentage contribution of some characteristic metal oxides in raw materials. OP100 presented higher MgO and CaO than Na_2_O content in ash, but comparing with OP0, OP100 presented high Na_2_O, K_2_O and SiO_2_ proportions. The total mineral contribution was greater in the case of OP100.

**Table 3:** Mineral ash composition of raw materials.

Sample	Na_2_O	MgO	Al_2_O_3_	SiO_2_	CaO	K_2_O	Fe_2_O_3_
OP100	0.97	4.68	2.51	22.28	4.01	43.98	0.71
OP0	0.65	11.96	11.14	2.50	15.76	14.46	1.25

**Figure 1:**
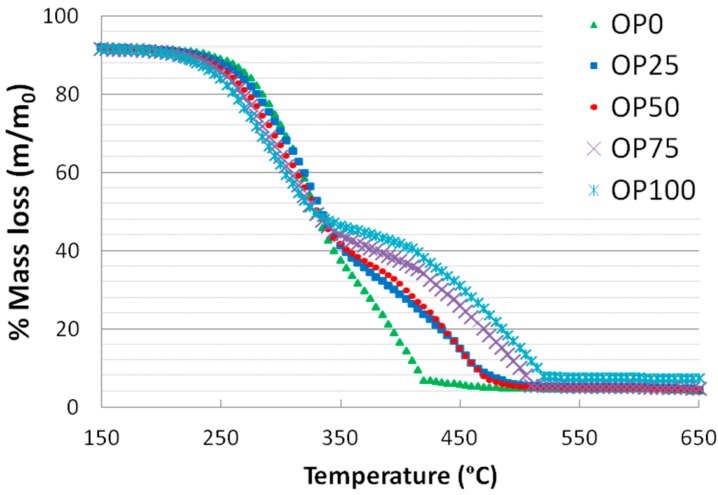
TG curves of different blends.

**Figure 2:**
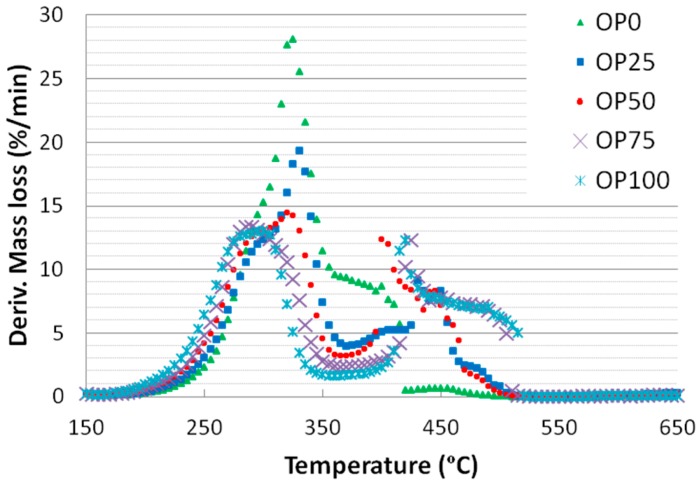
DTG curves of different blends.

Figure 1 and Figure 2 show the thermogravimetric (TG) and derivative thermogravimetric (DTG) profiles for the different blends, respectively. It is noticeable that the TG curves of the samples exhibit some differences: OP0 started its weight loss at a higher temperature than OP100 (245 against 215 °C, respectively). The mass loss associated to biomass thermal degradation is usually related to its lignocellulosic composition (hemicellulose, cellulose and lignin) [20]. In general, it is assumed that hemicellulose combustion is completed at around 350 °C, that of cellulose is accomplished in the range 250–500 °C, while lignin is degraded on a wide range of temperature (250–900 °C) [21,22]. The fact that OP100 presents a more marked weight loss in the range 215–250 °C could be associated to a higher proportion of hemicellulose. However, the fact that cellulose starts decomposing at a lower temperature could also be related to the differences on inorganic composition [23], since the high potassium content has been related to an enhancement of cellulose combustion (note that OP100 shows the highest proportion of potassium, Table 2 and Table 3) [24]. Moreover, the weight loss followed a continuous decrease in the case of OP0, whereas for the rest of the mixtures (from OP25 to OP100) there were two different slopes. The first weight loss of OP100 was more marked than that of OP0, probably due to its higher reactivity; on the other hand, the second weight loss of OP100 showed a softer slope. However, the relative contribution of hemicellulose might not be very important in any of the blends since the main peak does not show a defined shoulder.

Also, the thermal decomposition of olive pomace remained up to greater values of temperature, which could suggest that this precursor is composed by a higher proportion of any component that is harder to decompose such as lignin, whose decomposition temperature range is 250–900 °C [25].

**Figure 3:**
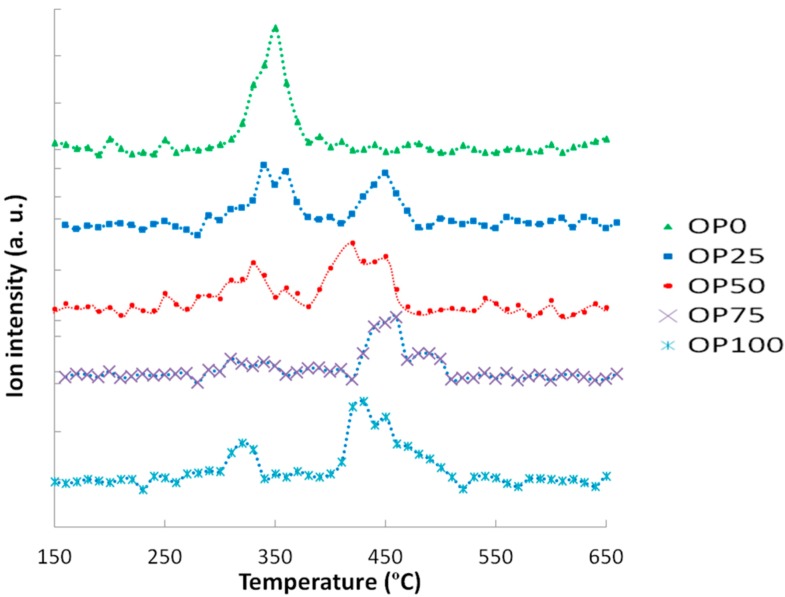
Mass spectra associated to benzene (*m*/*z* = 78).

**Figure 4:**
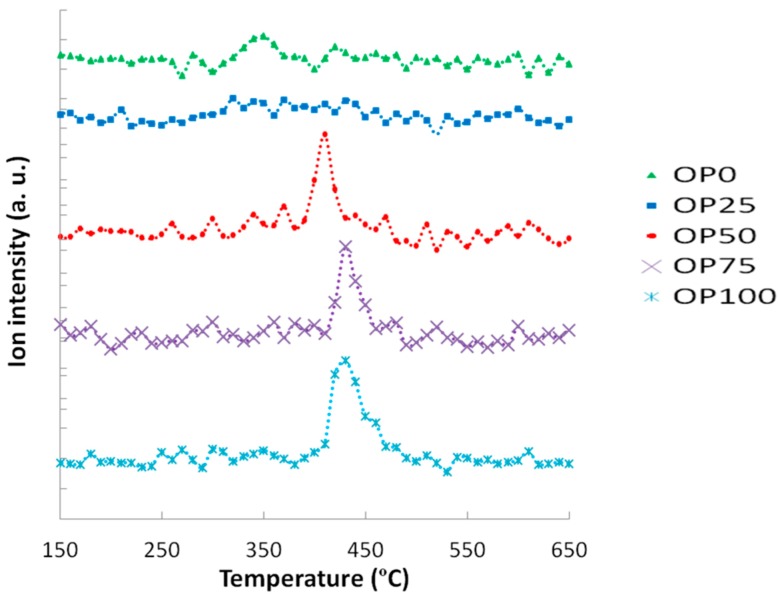
Mass spectra associated to toluene (*m*/*z* = 91).

These thermogravimetric characteristics can be related to the profiles found in the emissions of some of the compounds in the study. For instance, Figure 3 and Figure 4 show the mass spectra corresponding to benzene and toluene emissions, respectively. If we compare DTG and benzene emission curves, some similarities could be found. Thus, there are two peaks in the samples with a higher content of olive pomace (OP75 and OP100), which imply two different stages. In accordance with TG analyses, this could be associated with the fact that during combustion these samples are decomposed in two stages. On the other hand, this trend decreased with PK content, resulting in just one peak observed for OP0, in accordance with the one-stage thermal degradation in this temperature range. However, in the case of toluene, the first emission peak disappeared even for high OP contents.

Apart from the shape of the TG and DTG curves, it must be pointed out that the thermal release of these aromatic compounds was located at temperatures below 580 °C, and could therefore be associated to the carbon containing volatile matter [16], rather than the charring process.

**Figure 5:**
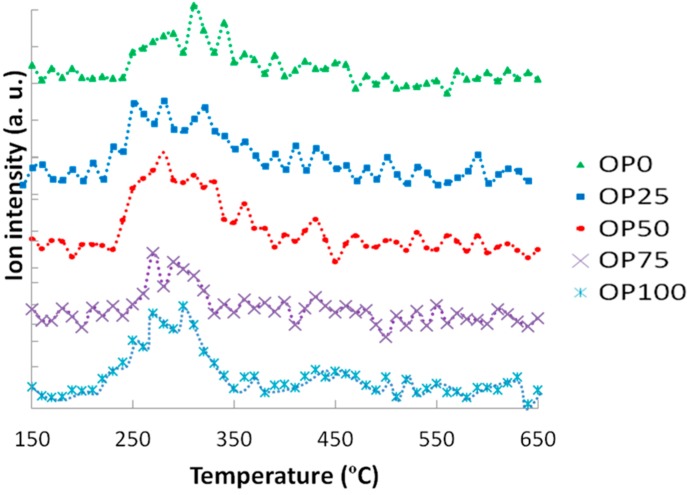
Mass spectra associated to sulphur dioxide (*m*/*z* = 64).

**Figure 6:**
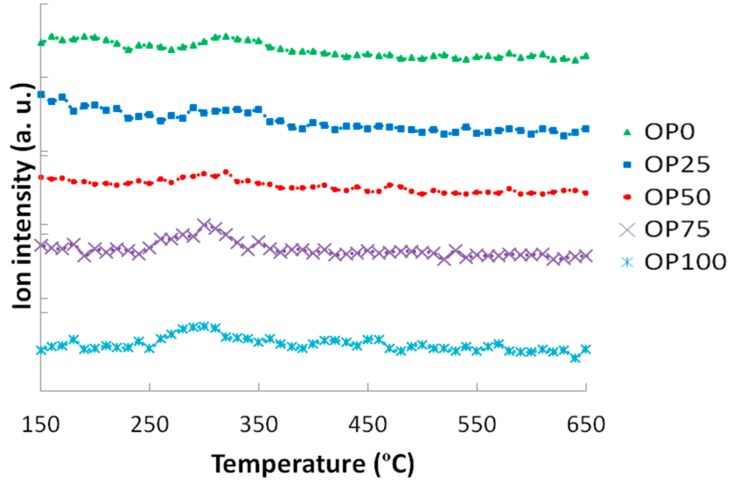
Mass spectra associated to hydrochloric acid (*m*/*z* = 36).

Figure 5 and Figure 6 show the mass spectra corresponding to the sulphur and chlorine compounds in the study; namely sulphur dioxide and hydrochloric acid, respectively. Two slightly higher SO_2_ peaks were observed for OP50 and OP100. If we compare these results with the elemental analyses in Table 2, it can be noticed that sulphur proportion did not exhibit great differences, nor a defined trend. Although previous works show evidence of the relationship between sulphur content and its associated emissions [11,16], it was not observed in this case probably because of the minimum differences in S content among the samples in study. Moreover, it should be noted that the ion intensities were so low that the differences might not be significant, as in the case of hydrochloric acid, whose peak emissions were quite similar among the blends in the study, although there were considerable differences in Cl content for the samples (Table 2). Additionally, chlorine could have led to another emission compound with higher mass/charge ratio, not detectable with the technique that was used in this study. Thus, alternative techniques (such as gas chromatography) would be suitable for this case.

**Figure 7:**
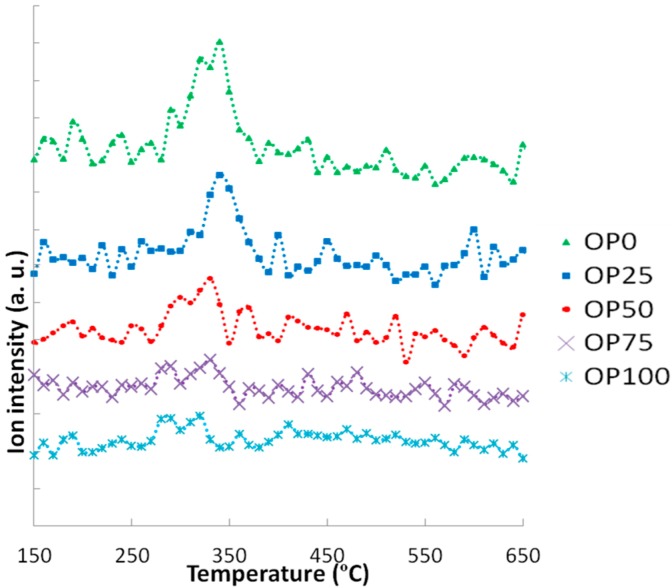
Mass spectra associated to 1,4-dioxin (*m*/*z* = 84).

Figure 7 shows the MS profiles corresponding to C_4_H_4_O_2_. Although all signals presented very low intensities, close to the determination limit of the mass spectrometer, some effects can be described. It seems that a higher OP content promoted the release of 1,4-dioxin at lower temperatures, while the addition of PK caused a shift of the maximun peak towards higher temperatures. Moreover, these peaks tended to be higher as PK content increases.

**Figure 8:**
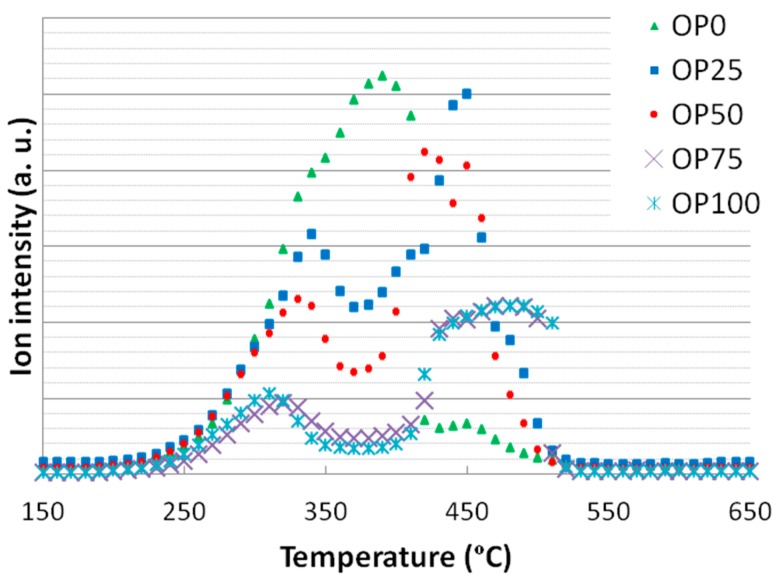
Mass spectra associated to carbon dioxide (*m*/*z* = 44).

The mass spectrometry intensity for *m*/*z* of 44 was assigned for the release of carbon dioxide and has been plotted in Figure 8. As observed, carbon dioxide was released at several stages: first, a wide peak centered at 350 °C was found, which might be associated to hemicellulose and cellulose decomposition, without misestimating a certain contribution of lignin. This peak was moved towards higher temperatures and got greater intensity with increasing OP content, confirming the higher relation to ligning thermal degradation. These two features confirmed the higher reactivity of this precursor. Some similarities between TG (Figure 1) events and CO_2_ MS analyses were found, as in the case of OP75 and 100, whose second peak was shifted towards 500 °C, showing a more marked slope (Figure 8). OP0 showed one single peak with one shoulder, possibly due to overlapping.

**Figure 9:**
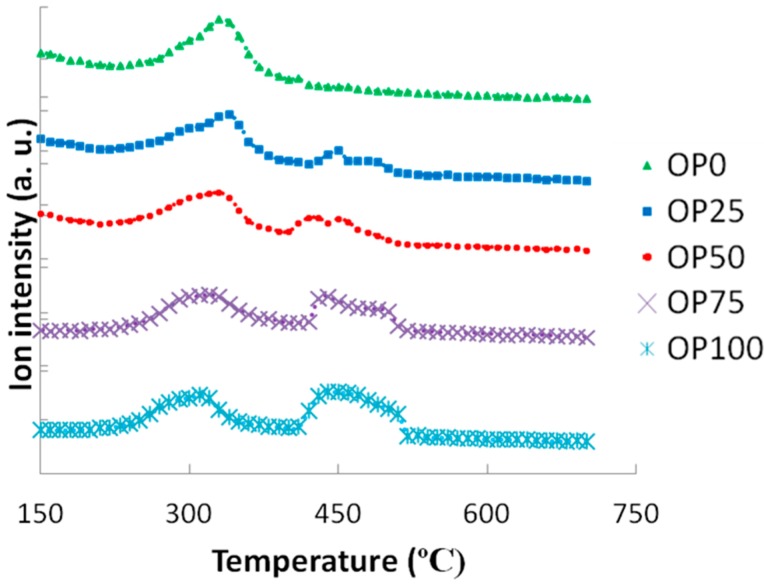
Mass spectra associated to nitrogen oxides (*m*/*z* = 30).

When it came to nitrogen oxides (Figure 9), again, some similarities can be observed between NO_x_ emissions and DTG curves. Thus, for low olive pomace proportion one single peak appearing at 330 °C was shown whereas for higher contents a second peak at a wide range (at around 450 °C) was observed. With the first peak emission being similar in height for all samples, the second one increased as N content was higher (that is, as olive pomace content increased). Consequently, it could be said that there was a correlation between nitrogen oxide emissions and its precursor, especially for the second emission step.

Finally, from the values of High Heating Value of the different blends prepared (in Table 1), it can be inferred that increasing OP content confers a greater energy content to the blends.

Thus, from an energetic point of view adding PK would not be interesting. However, if we take into account the emissions, it is clear that decreasing the OP proportion would make the process more environment-friendly.

## 3. Materials and Methods

Olive waste (olive pomace) and pyrenean oak residues were provided by local manufacturers. These residues were characterized in terms of their proximate analysis following the technical specifications CEN/TS 1474-2, CEN/TS 15148 and CEN/TS 14775, for moisture, volatile matter and ash, respectively [26]. The ultimate analysis was made with an elemental analyzer (Eurovector EA 3000, Eurovector, Milan, Italy), according to the norm CEN/TS 15104 (for determining the content of C, H and N) and CEN/TS 15289 (for S) [26]. For Cl, Na and K, analyses were carried out using ion chromatography.

High heating value (HHV) of the biomass residues was determined by a calorimetric bomb (Parr 1351) following the norm UNE164001 EX [27].

The densified pellets were prepared using a GR150E2 pelletizer, following the method described elsewhere [24]. The pellets were made with different proportions of wood and olive pomace (0%, 25%, 50%, 75% and 100%). The nomenclature used in all cases was OPX where X refers to the proportion of olive pomace. For example, OP25 represents a sample that has a 25% content in olive pomace and 75% in pyrenean oak.

Ash chemical composition of both pristine precursors was determined by Inductively Coupled Plasma Emission Spectroscopy (ICPES, Perkin-Elmer 2100, Waltham, MA, USA).

Thermogravimetry (TG) and mass spectroscopy (MS) analyses were carried out using a coupled TG–MS equipment (Thermogravimetric system, TA instruments, New Castle, DE, USA; Mass Spectrometer, Pfeiffer Tecnovac Thermostar GDS301 T3, Tecnovac, Alcobendas, Spain). The gas line between the TG and MS was heated to 200 °C in order to avoid cold points and prevent condensation of some of the gaseous products. The mass spectrometer signals were assigned to the following gaseous species, according to their maximum *m*/*z* value: (a) aromatic compounds: C_6_H_6_: *m*/*z* = 78 and C_7_H_8_: *m*/*z* = 91; (b) sulphur dioxide: SO_2_: *m*/*z* = 64; (c) hydrochloric acid: HCl: *m*/*z* = 36; (d) dioxin: C_4_H_4_O_2_: *m*/*z* = 84; (f) CO_2_: *m*/*z* = 44; and (g) NO_x_: *m*/*z* = 30. Ion intensity (in arbitrary units, a.u.) versus temperature was used in order to represent ion intensity evolution. Even though *m*/*z* signals might be shared with many evolved compounds (especially for benzene and sulphur dioxide), we have chosen the most representative *m*/*z* signal for each compound in study. An initial mass of 15.0 ± 0.1 mg was used, employing air as carrier gas (100 cm^3^·min^−1^) and a heating rate of 20 °C·min^−1^. The analyses were made at the temperature range 25–750 °C.

In order to guarantee reproducibility of results each run was made three times and the signals were normalized to the initial mass of the sample. Thus, it was possible to compare the peak height of the same compound evolved from different samples.

## 4. Conclusions

A study about emissions during biomass conversion was carried out, in order to assess the suitability of blending it with other less-pollutant products. With this purpose, TG coupled to MS was used to determine both emissions and weight loss events, along with proximate, ultimate analysis and ash composition to support data.

Reactivity of the samples increased along with increased OP content of the mixture. The different patterns found in TG and DTG profiles were related to the emissions evolved, analyzed by MS. Among the studied emissions, aromatics were associated with the range of temperature related to volatile decomposition (250–500 °C), with this range being narrower for lower OP contents. Nitrogen dioxide emissions were increased when the content of its precursor (N) in the blend composition was higher, whereas this effect was not observed for S and Cl, possibly due to its almost negligible differences in the raw materials or an alternative compound formation (not covered in this study), respectively. 1,4-Dioxin emissions were observed during the first stage, which is highly related to DTG curves. Consequently, OP0 and 25 had relatively high emissions, whereas curves of higher OP content exhibited a shift towards lower temperatures.

The analysis of the emissions was valued in comparison with the energetic content of the blends, and a compromise situation was found. Thus, in some cases, emission reduction was effective when pyrenean oak was added, as in the case of NO_x_ and toluene emissions. However, high heating value was reduced.

## References

[1] Patil V., Tran K., Giserod H.R. (2008). Towards sustainable production of biofuels from microalgae. Int. J. Mol. Sci..

[2] Cambero C., Sowlati T. (2014). Assessment and optimization of forest biomass supply chains from economic, social and environmental perspectives—A review of literature. Renew. Sustain. Energy Rev..

[3] Ferreira A.F., Ortigueira J., Alves L., Gouveia L., Moura P., Silva C. (2013). Biohydrogen production from microalgal biomass: Energy requirement, CO_2_ emissions and scale-up scenarios. Biorecour. Technol..

[4] Rabaçal M., Fernandes U., Costa M. (2013). Combustion and emission characteristics of a domestic boiler fired with pellets of pine, industrial wood wastes and peach stones. Renew. Energy.

[5] Miranda T., Esteban A., Rojas S., Montero I., Ruiz A. (2008). Combustion analysis of different olive residues. Int. J. Mol. Sci..

[6] Pazó J.A., Granada E., Saavedra A., Eguía P., Collazo J. (2010). Biomass thermogravimetric analysis: Uncertainty determination methodology and sampling maps generation. Int. J. Mol. Sci..

[7] Thomas V.M., McCreight C.M. (2008). Relation of chlorine, copper and sulphur to dioxin emission factors. J. Hazard. Mater..

[8] Amaral S., Carvalho J.A., Costa M., Soares Neto T.G., Dellani R., Leite L. (2014). Comparative study for hardwood and softwood forest biomass: Chemical characterization, combustion phases and gas and particulate matter emissions. Bioresour. Technol..

[9] Lusini I., Pallozzi E., Corona P., Ciccioli P., Calfapietra C. (2014). Novel application of a combustion chamber for experimental assessment of biomass burning emission. Atmos. Environ..

[10] Jiménez S., Ballester J. (2005). Influence of operating conditions and the role of sulfur in the formation of aerosols from biomass combustion. Combust. Flame.

[11] Chagger H.K., Kendall A., McDonald A., Purkashanian M., Williams A. (1998). Formation of dioxins and other volatile organic compounds in biomass combustion. Appl. Energy.

[12] Williams A., Jones A.M., Ma L., Pourkashanian M. (2012). Pollutants from the combustion of solid biomass fuels. Progr. Energy Combust. Sci..

[13] Miranda T., Arranz J.I., Montero I., Román S., Rojas C.V., Nogales S. (2012). Characterization and combustion of olive pomace and forest residue pellets. Fuel Process. Technol..

[14] Statheropoulos M., Kyriakou S., Tzamtzis N. (1998). Performance evaluation of a TG/MS system. Thermochim. Acta.

[15] Amorim E.B., Carvalho J.A., Soares Neto T.G., Anselmo E., Saito V.O., Dias F.F., Santos J.C. (2013). Influence of specimen size, tray inclination and air flow rate on the emission of gases from biomass combustion. Atmos. Environ..

[16] Skodras G., Palladas A., Kaldis S.P., Sakellaropoulos G.P. (2007). Cleaner co-combustion of lignite-biomass-waste blends by utilising inhibiting compounds of toxic emissions. Chemosphere.

[17] Ulloa C.A., Gordon A.L., García X.A. (2009). Thermogravimetric study of interactions in the combustion of blends of coal with radiata pine sawdust. Fuel Process. Technol..

[18] Shawalliah Idris S., Abd Rahman N., Ismail K., Bahari Alias A., Abd Rashid Z., Jindra Aris M. (2010). Investigation on thermochemical behaviour of low rank Malaysian coal, oil palm biomass and their blends during pyrolysis via thermogravimetric analysis (TGA). Bioresour. Technol..

[19] Menya E., Olwa J., Hangström P., Okure M. (2014). Assessment of pollution levels resulting from biomass gasification. J. Environ. Chem. Eng..

[20] Lv D., Xu M., Liu X., Zhan Z., Li Z., Yao H. (2010). Effect of cellulose, lignin, alkali and alkaline earth metallic species on biomass pyrolysis and gasification. Fuel Process. Technol..

[21] Otero M., Díez C., Calvo L.F., García A.I., Morán A. (2002). Analysis of the co-combustion of sewage sludge and coal by TG–MS. Biomass Bioenergy.

[22] Yang H., Yan R., Chen H., Zheng C., Lee D.H., Liang D.T. (2006). Influence of mineral matter on pyrolysis of palm oil wastes. Combust. Flame.

[23] Asadieraghi M., Wan Daud W.M.A. (2014). Characterization of lignocellulosic biomass thermal degradation and physiochemical structure: Effects of demineralization by diverse acid solutions. Energy Convers. Manag..

[24] Miranda M.T., Arranz J.I., Rojas S., Montero I. (2009). Energetic characterization of densified residues from Pyrenean oak forest. Fuel.

[25] Gani A., Naruse I. (2007). Effect of cellulose and lignin content on pyrolysis and combustion characteristics for several types of biomass. Renew. Energy.

[26] (2004). Biomass Standards. Technical Specifications CEN/TS-Solid Biofuels.

[27] (2005). Biocombustibles sólidos. Método para la determinación del poder calorífico.

